# An Evidence-Based Guideline for Surveillance of Patients after Curative Treatment for Colon and Rectal Cancer

**DOI:** 10.3390/curroncol29020062

**Published:** 2022-01-30

**Authors:** Erin Kennedy, Caroline Zwaal, Tim Asmis, Charles Cho, Jacqueline Galica, Alexandra Ginty, Anand Govindarajan

**Affiliations:** 1 Mount Sinai Hospital, 600 University Ave., Toronto, ON M5G 1X5, Canada; Anand.Govindarajan@sinaihealth.ca; 2 Program in Evidence-Based Care, McMaster University, 1280 Main St. W, Hamilton, ON L8S 4L8, Canada; 3 Ottawa Hospital Cancer Centre, The Hospital General Campus, 501 Smyth Rd., Ottawa, ON K1H 8L6, Canada; tasmis@toh.ca; 4 Southlake Regional Cancer Centre, 596 Davis Dr., Newmarket, ON L3Y 2P9, Canada; ccho@southlakeregional.org; 5 Queen’s University Cancer Research Institute, Division of Cancer Care and Epidemiology, Queen’s University, 92 Barrie Street, Kingston, ON K7L 3N6, Canada; jacqueline.galica@queensu.ca; 6 Dorval Medical FHT, 465 Morden Rd., Oakville, ON L6K 3W6, Canada; AGINTY@dorvalmedical.ca

**Keywords:** colorectal cancer, surveillance, follow-up, survivorship

## Abstract

Objective: To provide recommendations for a surveillance regimen that leads to the largest overall survival benefit for patients after curative treatment for Stage I–IV colon and rectal cancer. Methods: Consistent with the Program in Evidence-Based Care’s standard approach, guideline databases, i.e., MEDLINE, EMBASE, PubMed, Cochrane Library, and PROSPERO, were systematically searched. Then, we drafted recommendations and methodology experts performed an internal review of the resulting draft recommendations, which was followed by an external review by targeted experts and intended users. Results: Four systematic reviews and two randomized controlled trials were identified that provided evidence for recommendations. Conclusions: For patients with stage I–III colon cancer, a medical history and physical examination should be performed every six months for three years; computed tomography (CT) of the chest-abdomen-pelvis (CT CAP) should be performed at one and three years, or one CT CAP could be performed at 18 months; the use of carcinoembryonic antigen (CEA) is optional if CT imaging is being performed; and surveillance colonoscopy should be performed one year after the initial surgery. The frequency of subsequent surveillance colonoscopy should be dictated by previous findings, but generally, colonoscopies should be performed every five years if the findings are normal. There was insufficient evidence to support these recommendations for patients with rectal cancer, Stage IV colon cancer, and patients over the age of 75 years. Patients should be informed of current recommendations and the treating physician should discuss the specific risks and benefits of each recommendation with their patients.

## 1. Introduction

In 2020, it was expected that approximately 26,900 Canadians would be diagnosed with colorectal cancer (Canadian Cancer Society) [1]. Recent studies have found five-year recurrence rates for patients who have curative surgery for colorectal cancer (CRC) are approximately 20–30% [2,3,4]. Recurrence may occur either locally or metastasize to other organs, most commonly the liver and/or lungs. CRC survivors compose one of the largest groups of cancer survivors, and therefore, optimal follow-up care for this population is important, given the significant effects not only on patient care, but also health resource allocations. The principal aims of follow-up programs are to detect early recurrence in order to improve survival and quality of life while minimizing costs and harm from unnecessary tests. Many CRC patients in whom early recurrence is detected are eligible for a second curative intent surgery, for which the survival rate is approximately 20% [2]. Therefore, determining whether early detection leads to improved overall survival and the optimal regimen is key to establishing the effectiveness of a CRC surveillance program.

Currently, a combination of tests is used to assess early recurrence, and these tests most commonly include: CT chest-abdomen-pelvis (CT CAP), CEA, and colonoscopy. These tests are directed to areas of potential disease and conducted at pre-established intervals based on the incidence of recurrent disease that occurs at an exponential rate over the first two years and less frequently thereafter. While the current Ontario guidelines recommend higher-intensity follow-up for patients with CRC, more recently, there has been increasing evidence comparing higher and lower intensity regimens. 

The Program in Evidence-Based Care (PEBC) of Ontario Health, Cancer Care Ontario (OH (CCO)) works with Ontario stakeholders to develop evidence-based and evidence-informed guidance documents using the methods of the Practice Guidelines Development Cycle [5,6] and the AGREE II framework [7] as a methodological strategy for guideline development. This process includes a systematic review, interpretation of the evidence, and the development of draft recommendations by the Working Group, as well as internal reviews by content and methodology experts, and external reviews by Ontario clinicians, patient representatives, and other stakeholders.

The purpose of this guideline was to perform a review of systematic reviews as well as new evidence, and to develop evidence-based recommendations for a surveillance regimen for patients with curative intent treatment. This will build upon the previous guideline [8] and create a document that synthesizes the evidence, while taking into consideration patient information and support needs. This paper focuses on clinical outcomes and is part of a larger PEBC guideline that provides recommendations regarding overall survival, quality of life, patient informational needs, and models of care. The full guideline can be found at the OH (CCO) website: https://www.cancercareontario.ca/en.

### 1.1. Research Question

What is the surveillance regimen that has the best overall survival for patients after curative intent treatment for Stage I–IV colon and rectal cancer?

### 1.2. Intended Users

Intended users of the guideline include clinicians (e.g., medical oncologist, radiation oncologist, surgeon, advanced practice nurse, physician assistant, primary care providers (family physician, nurse practitioner, and family practice nurse)) involved in the delivery of care for colorectal cancer survivors and health care organizations and system leaders responsible for offering, monitoring, or providing resources for colorectal cancer survivorship protocols.

## 2. Materials and Methods

This review was conducted in two planned stages, including a search for systematic reviews followed by a search for new primary literature. These stages are described in the subsequent sections. 

### 2.1. Literature Search

A search for existing guidelines using the search terms: colorectal, survivors, and follow-up, was undertaken to determine whether any guideline could be endorsed. Evidence-based guidelines with systematic reviews published after 2018 were included if the guideline had a score of 5/7 or above on the Rigor of Development section of the AGREE II [7]. This guideline is an update; therefore, the search dates and the search terms were similar to those used in the original guideline work.

A search was conducted for existing systematic reviews on 1 May 2019 and a search for primary literature was conducted on 5 June 2019. The databases searched were OVID MEDLINE, EMBASE, and the Cochrane Database of Systematic Reviews. Search terms can be found in Appendix A. An update for the literature search was completed September 2020. 

Systematic reviews were included if they were in English and relevant to the research question. Primary articles were selected for inclusion if they were randomized controlled trials with a minimum follow-up of two years and the population consisted of patients with colorectal cancer whose primary treatment was with curative intent and were without evidence of disease. Articles were excluded if they were letters, comments, editorials, non-English publications, abstracts, or published before 2011. 

All reviews and primary studies that met the inclusion criteria underwent data extraction by CZ, with all extracted data and information audited subsequently by an independent auditor. Ratios, including HRs, were expressed with a ratio of <1.0 indicating benefit for the experimental group for a given outcome. Systematic reviews were assessed using the Risk of Bias (ROBIS) tool [9]. RCTs were assessed for quality and potential bias using Cochrane Risk of Bias tool (RoB) [10].

The certainty of the evidence per outcome for each comparison, taking into consideration risk of bias, inconsistency, indirectness, imprecision, and publication bias was assessed using the Grading of Recommendations, Assessment, Development and Evaluations (GRADE) framework [11].

### 2.2. Internal Review

The guideline was evaluated by the CCO Gastrointestinal Disease Site Group (GDG) Expert Panel and the PEBC Report Approval Panel (RAP). For the guideline to be approved, 75% of the Expert Panel must cast a vote indicating whether or not they approve the document, or abstain from voting for a specified reason, and of those that vote, 75% must approve the document. The PEBC RAP must also unanimously approve the document. A patient consultation group, consisting of patients, survivors, and/or caregivers, reviewed the draft recommendations and provided feedback on the comprehensibility, appropriateness, and feasibility to the working group’s health research methodologist. 

### 2.3. External Review

Peer reviewers were identified by the working group as clinical and/or methodological experts on the topic. Feedback was obtained through a brief online survey of healthcare professionals and other stakeholders who are the intended users of the guideline. All relevant surgeons (CRC, surgical oncology, and hepatobiliary), primary care physicians, radiologists and other imaging professionals, medical oncologists, nurses, and nurse practitioners in the PEBC database were contacted by email to inform them of the survey. 

## 3. Results

### 3.1. Literature Search Results

In total, 22 guidelines were found. None of the guidelines were deemed fully endorsable because the recommendations needed updating or were developed through consensus, and therefore, did not meet the inclusion criteria. 

There was a total of 3830 articles found through the original and updated literature search; 363 articles and 25 systematic reviews were selected for full-text review. Four systematic reviews [2,12,13,14] and two RCTs met the inclusion criteria and were retained [3,4] and were assessed for risk of bias. The risk of bias in the systematic reviews was considered low for each domain and overall. The RCTs were found to be of a low risk of bias (see Appendix B for PRISMA diagram, Appendix C for evidence summary tables, and Appendix D for quality assessment results). Overall, there was consensus within the working group that there was a high level of certainty in the evidence based on the GRADE framework.

### 3.2. Internal Review

Among the 15 members in the GDG Expert Panel, 13 members voted, and two members abstained, for a total of 87% response in December 2020. Among those that cast votes, 12 members approved the document (92%). Three RAP members reviewed this document in August 2020; it was approved in September 2020. Comments from the expert panel reflected the need to remove recommendations for stage IV patients due to insufficient evidence. RAP comments asked for additional clarity to make the recommendations more specific so that they could be easily applied into clinical practice by providers. The patient consultation group supported the patient-focused recommendations and suggested that patients’ families be included in communication recommendations.

### 3.3. External Review

Seven targeted peer reviewers from Ontario, British Columbia, and New York were identified by the working group and five agreed to be reviewers. The main comment was a request for additional clarity by making the recommendations about the surveillance regimen more specific so that they could be easily applied into clinical practice by providers to their individual patients. The online survey was sent to intended users of the guideline (n = 183) and fourteen responses (7.6%) were received. Feedback indicated approval but requested additional clarifications regarding the recommendations. The final guideline recommendations reflect the integration of feedback obtained through both internal and external review processes.

## 4. Recommendations, Key Evidence, and Interpretation of the Evidence

*Recommendations for Patients with Stage I–III Colon Cancer:*

- A medical history and physical examination should be performed every six months for three years.
- Computed tomography (CT) of the chest-abdomen-pelvis (CT CAP) should be performed at one and three years, or one CT CAP could be performed at 18 months.
- The use of carcinoembryonic antigen (CEA) is optional if CT imaging is being performed.
- Surveillance colonoscopy should be performed one year after the initial surgery. The frequency of subsequent surveillance colonoscopy should be dictated by the findings of the previous one, but it generally should be performed every five years if the findings of the previous one is normal (summary in Table 1).

*Qualifying Statements:*

- The use of CEA in combination with CT CAP does not lead to a survival advantage as compared with CT CAP alone.
- CEA is optional in patients with elevated CEA prior to treatment provided that CT CAP imaging is being performed.
- If complete colonoscopy was not performed in the course of diagnosis and staging (e.g., due to obstruction), a complete colonoscopy should be performed within six months of completing primary therapy.
- There was insufficient evidence to support these recommendations for patients with rectal cancer, patients with stage IV colon cancer, and patients over the age of 75 years. Therefore, the follow-up in those patients is at the discretion of the treating physician.
- There was no evidence to support follow-up in patients with stage I–III colon cancer beyond three years. Therefore, follow-up after this time period is at the discretion of the treating physician.
- These recommendations do not apply to patients with rectal cancer undergoing non-operative management or to patients with increased risk of cancer including but not limited to inflammatory bowel disease, familial adenomatous polyposis, and Lynch syndrome.
- Patients should be informed of these current recommendations and the treating physician should discuss the specific risks and benefits of these recommendations with their patient.

### 4.1. High- versus Low-Intensity Surveillance Regimens

A Cochrane review by Jeffery et al. [2], conducted in 2019, evaluated the outcomes associated with high- and low-intensity follow-up programs in patients with colorectal cancer treated with curative intent. This review included 19 RCTs comparing different follow-up strategies that included comparisons of follow-up as compared with no follow-up, follow-up strategies of varying intensity (e.g., differing frequency or quantity of testing or both), and follow-up in different healthcare settings (primary care vs. hospital) (see Appendix C). 

Among the 19 RCTS found, the review identified 15 RCTs and 12,528 patients that showed that there was no survival benefit for intensifying the follow-up regimen (overall survival hazard ratio (HR) of 0.91 (95% confidence interval (CI) 0.80 to 1.04) and colorectal-specific survival of 0.93 (95% CI 0.81 to 1.07)) [2]. 

This review also showed no difference in the detection of recurrence with more intensive follow-up regimens (relapse-free survival HR of 1.05 (95% CI 0.92 to 1.21)); however, significantly more surgical procedures for recurrence were performed in the higher intensity follow-up regimens (relative risk (RR) of 1.98 (95% CI 1.53 to 2.56)). These data also showed that 90% of the recurrences were found within 36 months of follow-up. A subgroup analysis showed that there was no difference in overall survival in studies using CEA versus no CEA, CT versus no CT, or more than two CT scans versus two or fewer CT scans [2].

The COLOFOL trial was a multicentre trial that randomized 2509 patients, treated for stage II and III colorectal cancer, to high-intensity follow-up consisting of a CEA at one month postoperatively followed by CEA and CT CAP at 6, 12, 18, 24, and 36 months, or low-intensity follow-up consisting of CEA at one month postoperatively followed by CEA and CT CAP at 12 and 36 months after surgery. The primary outcomes for this study were overall survival or cancer-specific recurrence and no difference was seen between the high- and low-intensity groups (risk difference of 1.1% (95% CI −1.6 to 3.8) and risk difference of 2.2% (95% CI, −1.0–5.4%), respectively). The secondary outcome for the trial was CRC specific recurrence and there was no difference found between the groups (risk difference of 2.2% (95% CI −1.0% to 5.4%, *p* = 0.15)). There were no significant differences in overall survival between cancer stages [3]. 

The FACS (Follow-up after Colorectal Surgery) trail was a multicentre trial that randomized 1202 patients treated for Dukes’ stage A–C cancer to minimum follow-up (CT CAP at 12 to 18 months if requested at study entry by the treating clinician) or one of three other higher intensity groups that included CEA and CT CAP combined (CEA every 3 months for 2 years, and then every 6 months for 3 years; CT CAP every 6 months for 2 years, and then annually for 3 years), CEA alone, or CT CAP alone. The results of this study showed that overall and disease-specific survival were similar between the minimum follow-up and higher intensity regimens. This study also showed that detection of recurrence at scheduled visits was higher in the higher intensity follow-up groups and this led to more surgical procedures for recurrence in the higher intensity follow-up groups. There were no differences in overall survival between groups for patients with Dukes’ A, B, or C [4]. 

In summary, the evidence consistently shows that there is no survival benefit for intensifying the surveillance regimen. While higher intensity programs did allow for earlier detection, this did not translate into an improved overall or cancer specific survival. 

### 4.2. Specific Modalities in Surveillance Regimen

While the Cochrane meta-analysis was comprehensive, this analysis did not evaluate or compare individual modalities. A subgroup analysis showed that there was no difference in overall survival in studies using CEA versus no CEA, CT versus no CT, or more than two CT scans versus two or fewer CT scans. 

Three systematic reviews evaluated the effectiveness of individual modalities [12,13,14]. One review evaluated CEA, CT, and colonoscopy [12], one review evaluated CEA only [13], and one review evaluated colonoscopy only [14]. 

#### 4.2.1. CT Scan

Pita-Fernández et al. performed a meta-analysis to compare high-intensity with low-intensity follow-up regimens using overall survival as the primary outcome [12]. This review included 11 RCTs (4055 patients). Overall survival was reported for individual diagnostic tests including CEA, CT, and colonoscopy (see Appendix C). The results showed that having a CT scan (vs. no CT scan) led to improved overall survival (HR of 0.80 (95% CI 0.66 to 0.98)). These results are different than the Cochrane subgroup analysis that showed no survival difference between CT versus no CT or more than 2 CT scans versus 2 or fewer CT scans. This is most likely because the systematic review by Pita-Fernandez was published before the Cochrane review and did not include some of the newer, larger RCT studies. 

The FACS RCT factorial analysis showed that having a CT scan led to an increase in recurrence detected by scheduled follow-up (15.3% vs. 7.3%, *p* < 0.001), but this did not lead to a significance difference in overall detection of recurrence (18.1% vs. 15.6%, *p* = 0.25 [4]. There was significantly more surgical treatment of recurrence in the CT vs. no CT group (8.2% vs. 4.5%, *p* = 0.009). This also did not translate into a significant difference in overall survival (25.8% vs. 25.1%, *p* = 0.79) or disease-free survival (13.8% vs. 14.3%, *p* = 0.92).

#### 4.2.2. CEA Test

The meta-analysis by Pita-Fernández et al. showed a trend toward improved survival with CEA (vs. no CEA) (HR of 0.73 (95% CI 0.51 to 1.05)), but this did not reach statistical significance [12]. 

Similarly, no significant differences between CEA and no CEA for recurrences detected by scheduled follow-up (12.5 vs. 10.2%, *p* = 0.21) or detection of overall recurrence was found by the FACS study factorial comparison (17.3% vs. 16.5%, *p* = 0.72). There was no difference in the rate of surgical salvage between the CEA vs. no CEA testing groups (6.6% vs. 6.0%, *p* = 0.65) [4]. 

A health technology assessment by Shinkins et al. included a meta-analysis of 52 studies and assessed the sensitivity and specificity of single and serial CEA testing for detection of cancer recurrence [13]. The results of the pooled analysis with a threshold of 5 μg/L used in 23 studies (4585 patients) showed a sensitivity of 71% (95% CI 64 to 76) and a specificity of 88% (95% CI 84 to 92). Therefore, for 1000 people tested, 14 cases of recurrence were detected, six cases were missed, and 118 people were referred unnecessarily for further testing.

#### 4.2.3. Colonoscopy

The meta-analysis by Pita-Fernández et al. showed that colonoscopy (vs. no colonoscopy) (HR of 0.65 (95% CI 0.53 to 0.81)) led to improved overall survival [12]. 

Fuccio et al. conducted a meta-analysis of 27 studies to examine the CRC detection rates and timing of CRC recurrence at anastomotic and non-anastomotic locations [14]. They found that the risk of CRC recurrence at anastomoses was significantly lower 24 months after resection than earlier; 70.5% of all CRC recurrences at the anastomosis were detected within 24 months of surgery and 90.8% within 36 months of surgery. The risk for CRC at non-anastomotic locations was significantly reduced more than 36 months after resection as compared with earlier and 53.7% of all non-anastomotic CRCs were detected within 36 months of surgery.

In the FACS trial, three luminal recurrences were detected in 601 (0.5%) patients at the two-year colonoscopy in the groups being monitored by CT imaging. Three new cancers were detected in 1202 patients (0.2%) (all groups) at the five-year colonoscopy [4]. 

The original PEBC guideline states that a postoperative colonoscopy should be performed one year following surgery. The frequency of subsequent surveillance colonoscopies should be dictated by the findings of this initial postoperative colonoscopy and, in general, should be performed a minimum of every five years [8]. However, if a complete colonoscopy was unable to be performed preoperatively, then a postoperative colonoscopy is recommended within six months of surgery. This original recommendation was based on the results of the National Polypectomy Study [15]. 

#### 4.2.4. Clinic Visits

The meta-analysis by Pita-Fernandez et al. showed that clinic visits (vs. no clinic visits) (HR, 0.57 and 95 % CI 0.0.35 to 0.92) led to improved overall survival [12]. 

### 4.3. Implementation Considerations

There was insufficient evidence to support these recommendations for patients with rectal cancer, patients with stage IV colon cancer, and patients over the age of 75 years. Likewise, there was no evidence to support follow-up in patients with stage I–III colon cancer beyond three years. The follow-up in such patients is at the discretion of the treating physician. 

These recommendations do not apply to patients with rectal cancer undergoing non-operative management or to patients with increased risk of cancer including but not limited to inflammatory bowel disease, familial adenomatous polyposis, and Lynch syndrome. Patients should be informed of these current recommendations and the treating physician should discuss the specific risks and benefits of these recommendations with their patient. At minimum, patients should be informed of current guideline recommendations and the treating physician should discuss the specific advantages and disadvantages with their patient based on the specific details of their case.

### 4.4. Further Research

Future studies to assess the effect of intensifying follow-up for rectal cancer, stage IV colon cancer, and patients over the age of 75 years as well as quality of life, harm, cost, resource utilization, patient preference for follow-up care, and racial disparities are warranted.

## 5. Discussion

The main finding of this review was that that there was relatively little benefit from intensifying surveillance in patients treated with curative intent for Stage I–III colon cancer and there was insufficient evidence to make recommendations for patients with rectal cancer, stage IV colon cancer, and patients over the age of 75 years. The recommendations based on this finding was developed through a review of the literature, development of recommendations based on the evidence by a working group of experts, review of the recommendations by methodological and clinical experts and patient representatives, and modifications based on that feedback.

While these results suggest that early detection does not lead to improved survival, it is important to note that surgery for recurrence may be effective in some patients, with five-year survival ranging from 25–37% [16]. A possible explanation for these findings is tumor biology. For example, on the one hand, finding an early or rapidly progressing recurrence with a high-intensity surveillance protocol in a patient with poor tumor biology may lead to earlier surgery, but this is unlikely to have any effect on overall survival since there is a high risk of developing further metastatic disease following surgery. Furthermore, complications from unnecessary surgery may delay subsequent treatment that may further impact survival. On the other hand, a patient with a recurrence that remains stable for several months (i.e., not detected early) is likely to have a lower risk of developing further metastasis and is more likely to be cured or have improved overall survival with surgery. Conversely, while the evidence for early detection and overall survival was quite robust, the number of patients undergoing surgery for curative intent included in the RCTs was relatively small, and therefore, underpowered to detect small but significant differences in overall survival and disease-specific survival.

While the working group would have liked to provide a stratified surveillance program for patients at “high” and “low” risk of recurrence, the evidence did not show any difference in detection of recurrence or overall survival between patients with Stage I–III colon cancer with either high intensity or low intensity follow-up regimens. An older study by Secco et al. [17], which used only CEA and ultrasound imaging as part of the follow-up regimen, defined high risk patients, as those who had a left sided Dukes B2 or higher colon, any low rectal cancer, a pretreatment CEA greater than 7.5 ng/mL, poorly differentiated grade and/or a mucinous or signet cell adenocarcinoma. High and low risk patients were both randomized to either risk-adapted follow-up or minimal follow-up. The results showed that the actuarial 5-year survival were higher in the risk adapted group for both high and low risk groups (high risk 50% versus 32%; low risk 80% versus 60%). Therefore, future studies to assess the risk-adapted surveillance regimens for patients and high and low risk of recurrence are highly relevant to validate these findings and tailor surveillance regimens based on prognostic indicators [18].

Sustained knowledge translation will be critical to implementation of these guidelines into clinical practice and practice variation in Ontario. Key components of this will need to include discussion around the systematic review findings that early detection of colorectal recurrence does not seem to lead to improved survival as well as issues including that “more” investigations may possibly expose patients to more harm and lead to overutilization of health resources. Knowledge translation activities will also focus on patient-physician communication to ensure that patients understand how these recommendations apply specifically to them as well as the risk and benefits of these recommendations. 

### Limitations

While the results of the Cochrane review were quite robust, one of the limitations of this study was that regimens were categorized as higher versus lower intensity regimens, and therefore, a higher intensity regimen in one study may have been similar to the lower intensity regimen in another study. Therefore, it was not possible to determine the optimal follow-up regimen based on these Cochrane results.

However, both the COLOFOL and FACS trials did directly compare specific follow-up regimens. The COLOFOL trial showed that CT CAP at 12 and 36 months postoperatively has similar overall survival to higher intensity follow-up with CT CAP at six, 12, 18, 24, 30, and 36 weeks postoperatively [3]. Similarly, the FACS trial showed that minimum follow-up with CT CAP at 12–18 months had similar overall survival to higher intensity follow-up regimens including CEA only, CT only, and CEA and CT over a five-year follow-up period [4].

Furthermore, there were very few RCT identified during the literature review that evaluated the use of abdominal ultrasound and/or chest X-ray in higher and lower intensity surveillance regimens, and therefore, it was not possible to provide recommendations regarding these modalities. 

While there did not seem to be any differences in quality of life or harm between the regimens, these data were extremely limited. Similarly, the number of rectal cancer and stage IV patients and racialized patients included in these studies was extremely small, and therefore, these results cannot be generalized to these groups at this time. While quality of life and related issues were researched in the full guideline, these outcomes were beyond the scope of this paper, and will be published separately.

Lastly, while there was a very robust internal and external review process, there was a very low response rate (7.6%) to an online survey to intended users to provide feedback on the guideline. This may have implications on the generalizability and uptake of this guideline.

## 6. Conclusions

There is an increasing body of evidence that early detection provided by intensifying follow-up regimens for colorectal cancer does not lead to improved overall or disease-specific survival. Therefore, use of lesser intensity follow-up regimens is reasonable. Further studies are warranted to assess the effect of intensifying follow-up for rectal cancer and stage IV patients as well as quality of life, harm, cost, resource utilization, patient preference for follow-up care, and racial disparities.

## Figures and Tables

**Table 1:** Recommended evaluation and intervals for routine surveillance of stage I–III colon cancer survivors.

Intervention	Interval	
	Years 1 to 3	Years 4 and 5
Physical examination	Every 6 months	At discretion of treating physician
CEA	At discretion of treating physician	At discretion of treating physician
CT of the chest- abdominal-pelvic imaging (CT CAP)	CT CAP at years 1 and 3 OR CT CAP at 18 months	At discretion of treating physician
Colonoscopy	At 1 year following surgery, the frequency of subsequent surveillance colonoscopies should be dictated by the findings of the previous one but, in general, a colonoscopy should be performed every 5 years if the findings of the previous one are normal.	

Abbreviations: CEA, carcinoembryonic antigen; CT, computed tomography.

**Figure A1:**
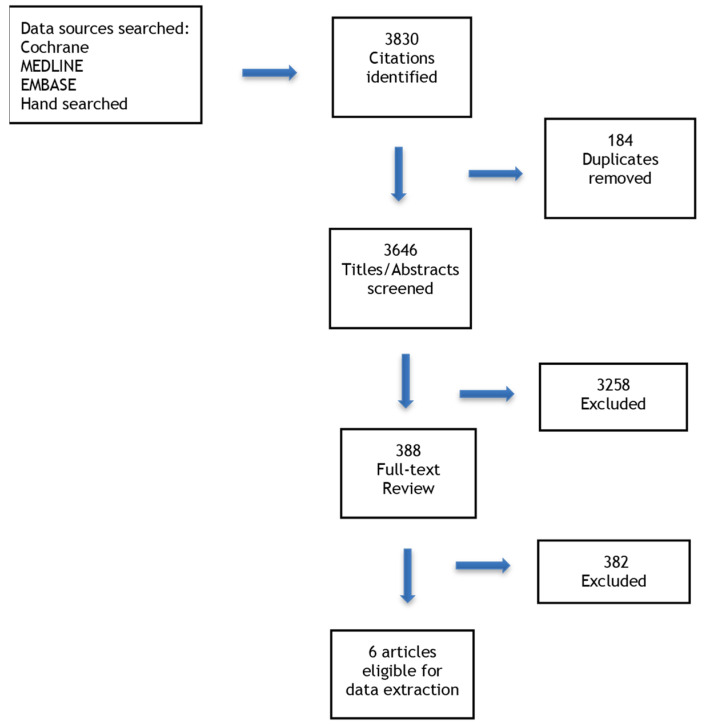
Primary literature results plus updated search results.

## Data Availability

Not applicable.

## Appendix A. Literature Search Strategy

- **MEDLINE**
  1. exp colorectal neoplasms/
  2. colorectal cancer:.mp.
  3. rectal cancer:.mp.
  4. CRC:.mp.
  5. or/1–4
  6. surveillance:.mp.
  7. follow-up:.mp.
  8. survivor:.mp.
  9. prevent:.mp.
  10. (late adj2 effect:).mp.
  11. or/6–10
  12. 5 and 11
  13. recurrence/
  14. neoplasm recurrence, local/
  15. recurren:.mp.
  16. or/13–15
  17. 12 and 16
  18. limit 17 to (english language and humans)
  19. limit 18 to yr = “2011–current”
  20. meta-analysis.pt.
  21. meta-analy$.tw.
  22. metaanal$.tw.
  23. (systematic adj (review$1 or overview$1)).tw.
  24. meta-analysis as topic/
  25. or/20–24
  26. cochrane.ab.
  27. (cinahl or cinhal).ab.
  28. embase.ab.
  29. scientific citation index.ab.
  30. bids.ab.
  31. cancerlit.ab.
  32. or/26–31
  33. reference list$.ab.
  34. bibliograph$.ab.
  35. hand-search$.ab.
  36. relevant journals.ab.
  37. manual search$.ab.
  38. or/33–37
  39. selection criteria.ab.
  40. data extraction.ab.
  41. 39 or 40
  42. review.pt.
  43. review literature as topic/
  44. 42 or 43
  45. 41 and 44
  46. comment.pt.
  47. letter.pt.
  48. editorial.pt.
  49. or/46–48
  50. 25 or 32 or 38 or 45
  51. 50 not 49
  52. practice guideline/
  53. practice guideline$.mp.
  54. 52 or 53
  55. 51 or 54
  56. 19 and 55
  57. 19 not 49
  58. (comment or letter or editorial or note or erratum or short survey or news or newspaper article or patient education handout or case reports or historical article).pt.
  59. 19 not 58
  60. 59 and 55
  61. 59 not 55
  62. case series.mp.
  63. 61 not 62
  64. 59 not 62
- **EMBASE**
  1. exp colorectal cancer/ or exp colorectal carcinoma/ or exp colorectal tumor/ or exp colorectal tumour/
  2. colorectal cancer:.mp.
  3. rectal cancer:.mp.
  4. CRC:.mp.
  5. or/1–4
  6. surveillance:.mp.
  7. exp follow-up/
  8. after care/
  9. long term care/
  10. follow-up:.mp.
  11. survivor:.mp.
  12. prevent:.mp.
  13. (late adj2 effect:).mp.
  14. or/6–13
  15. 5 and 14
  16. exp recurrent cancer/ or exp recurrent disease/
  17. recurren:.mp.
  18. 16 or 17
  19. 15 and 18
  20. limit 19 to (human and english language)
  21. limit 20 to yr = “2011–current”
  22. exp meta-analysis/
  23. ((meta adj analy$) or metaanaly$).tw.
  24. (systematic adj (review$1 or overview$1)).tw.
  25. or/22–24
  26. cancerlit.ab.
  27. cochrane.ab.
  28. embase.ab.
  29. (cinahl or cinhal).ab.
  30. scientific citation index.ab.
  31. bids.ab.
  32. or/26–31
  33. reference list$.ab.
  34. bibliograph$.ab.
  35. hand-search$.ab.
  36. manual search$.ab.
  37. relevant journals.ab.
  38. or/33–37
  39. data extraction.ab.
  40. selection criteria.ab.
  41. 39 or 40
  42. review.pt.
  43. 41 and 42
  44. letter.pt.
  45. editorial.pt.
  46. 44 or 45
  47. 25 or 32 or 38 or 43
  48. 47 not 46
  49. exp practice guideline/
  50. practice guideline$.tw.
  51. 49 or 50
  52. 48 or 51
  53. 21 and 52

## Appendix C. Evidence Tables

**Table A1:** Results of Jeffery et al. meta-analysis, high- versus low-intensity follow-up.

Systematic Review	Outcomes	Number of Studies	Hazard Ratio	Heterogeneity	GRADE Assessment of Quality for Outcome
Jeffery, 2019 [2]	Overall survival	15 studies	HR 0.91, 95% CI 0.80 to 1.04	I^2^ = 18%, *p* = 0.25	High
	Colorectal cancer-specific survival	11 studies	HR 0.93, 95% CI 0.81 to 1.07	I^2^ = 0%, *p* = 0.57	Moderate
	Relapse-free survival	16 studies	HR 1.05, 95% CI 0.92 to 1.21	I^2^ = 41%, *p* = 0.05	High
	Salvage surgery	13 studies	RR 1.98, 95% CI 1.53 to 2.56	I^2^ = 31%; *p* = 0.14	High
	Symptomatic (interval) recurrences	7 studies	RR 0.59, 95% CI 0.41 to 0.86	I^2^ = 66%; *p* = 0.007	Moderate

Abbreviations: CI, confidence interval; HR, hazard ratio; RR = relative risk.

**Table A2:** Summary of study characteristics and results.

Evaluation	Study	Patients	Study Design	Outcomes	Results
Meta-analysis/HTAs					
CEA CT	Shinkins, 2017 [13]	52 studies plus re-analysis of FACS	Meta-analysis	Diagnostic accuracy of one test, trends and levels of CEA to trigger further investigation	Pooled analysis for 5 μg/L of 23 studies (4585 participants): Sensitivity 71% (95% CI 64% to 76%) Specificity 88% (95% CI 84% to 92%) Pooled analysis for 2.5 μg/L of 7 studies (1515 participants): Sensitivity 82% (95% CI 78% to 86%) Specificity 80% (95% CI 59% to 92%) Pooled analysis for 10 μg/L of 7 studies (2341 participants): Sensitivity 68% (95% CI 53% to 79%) Specificity 97% (95% CI 90% to 99%)
					In the secondary analysis of FACS data at 5 μg/L, Sensitivity 50% (95% CI 40% to 60%) Specificity (%) 93.3 (91% to 95%) Positive predictive value: 62% (51% to 72%) Negative predictive value: 90% (87% to 92%)
Colonoscopy CEA CT Clinic Visits	Pita-Fernandez 2015 [12]	11 studies with 4055 patients Curative surgery 1995–June 2014	Meta-analysis	Intensive strategies: overall survival, recurrence, evaluate diagnostic tests	Overall Survival Colonoscopy: 8 studies, HR = 0.75 (0.64–0.87) 4 studies comparing with less vs. more: HR = 0.86 (0.69–1.06) 4 studies with vs. without colonoscopy: HR = 0.65 (0.53–0.81) CEA testing: Total studies, 4 HR = 0.69 (0.52–0.93) Studies with less vs. more CEA: 1 HR = 0.57 (0.35–0.92) Studies with vs. without CEA: 3 HR = 0.73 (0.51–1.05) * includes FACS CT: Total studies, 6 HR = 0.80 (0.66–0.98) Studies with less vs. more CT: 0 Studies with vs. without CT, 6 HR = 0.80 (0.66–0.98) * includes FACS Clinic visits: Total studies, 3 HR = 0.59 (0.46–0.75) Studies with less vs. more CV: 2 HR = 0.59 (0.44–0.79) Studies with vs. without CV: 1 HR = 0.57 (0.35–0.92)
Colonoscopy	Fuccio, 2019 [14]	15,589 stage I-IV patients from 27 studies that used colonoscopy for surveillance after curative CRC surgery 1986–2017 The mean length of follow-up: 18–108 months	Meta-analysis	Primary outcomes were rates and timing of CRCs at anastomotic and non-anastomotic location.	296 non-anastomotic CRCs were detected over more than 16 years: cumulative incidence, 2.2% of CRCs; (95% CI 2–3%) risk of CRC at a non-anastomotic location was significantly reduced more than 36 months after resection compared with before this time point (non- anastomotic CRCs at 37–48 months vs. 6–12 months after surgery, OR = 0.61, 95% CI 0.37–0.98, *p* = 0.031) 53.7% of all non-anastomotic CRCs were detected within 36 months of surgery. 158 CRCs were detected at anastomoses over more than 16 years: cumulative incidence; 2.7% of CRCs; (95% CI: 2–4%) risk of CRCs at anastomoses was significantly lower 24 months after resection than before: CRCs at anastomoses at 25–36 months after surgery vs. 6–12 months, OR = 0.56, 95% CI 0.32–0.98, *p* = 0.036) 90.8% of all CRCs at anastomoses were detected within 36 months of surgery.
Randomized Controlled Trials					
Colonoscopy CEA CT	Wille-Jørgensen, 2018 [3]	2509 patients with stage II or III CRC resection	RCT	To assess the effect of scheduled measurement of CEA and CT as follow-up to detect recurrent CRC	Study Design: High-frequency group: CEA and CT CAP at 6, 12, 18, 24, and 36 months after surgery, n = 1253 patients. Low-frequency group: CEA and CT CAP at 12 and 36 months after surgery, n = 1256 patients Results: The 5-year overall patient mortality rate: high vs. low 13.0% (161/1253) vs. 14.1% (174/1256) (risk difference, 1.1% *p* = 0.43) The 5-year colorectal cancer–specific mortality rate: high vs. low frequency, 10.6% (128/1248) vs. 11.4% (137/1250) (risk difference 0.8%, *p* = 0.52) The colorectal cancer–specific recurrence rate: high vs. low frequency: 21.6% (265/1248) vs. 19.4% (238/1250) (risk difference 2.2%, *p* = 0.15)
Colonoscopy CEA CT	Mant, 2017 Primrose, 2015 FACS [4]	1202 patients with CRC resection	RCT	To assess the effect of scheduled measurement of CEA and CT as follow-up to detect recurrent CRC	Study Design: CEA only follow-up: CEA q 3 months for 2 years, then q 6 months for 3 years with single CT CAP at 12 to 18 months CT only follow-up: CT CAP q 6 months for 2 years and then annually for 3 years CEA and CT follow-up: CEA and CT CAP as per Group 1 and 2 Minimum follow-up: No scheduled follow-up except a single CT CAP at 12 to 18 months Results: Two-thirds of recurrences (134, 66.0%) were detected by a scheduled follow-up investigation: 87 (64.9%) by CT; 43 (32.1%) by CEA measurement. More recurrences were detected in the CT arm than in the CEA testing arm (9.4% vs. 6.3%; *p* = 0.16). The factorial comparison showed a significant absolute benefit only for CT (absolute difference 3.7%; *p* = 0.01). COL detected: 3 local recurrences of rectal tumours; 3 synchronous tumours; 2 metachronous tumours; low-risk adenomas in 76 patients (20.7%, n = 367); high-risk adenomas in 22 patients (5.9%, n = 367).

Abbreviations: CAP, chest-abdomen-pelvis; CEA, carcinoembryonic antigen; CI, confidence interval; COL, colonoscopy; CRC, colorectal cancer; CT, computed tomography; FACS, follow-up after colorectal surgery; HR, hazard ratio; HTA, health technology assessment; OR, odds ratio; q, measured; RCT, randomized controlled trial.

## Appendix D. Quality Assessment Scores

**Table A3:** ROBIS, systematic review/meta-analysis.

Study	Domain 1: Study Eligibility Criteria	Domain 2: Identification and Selection of Studies	Domain 3: Data Collection and Study Appraisal	Domain 4: Synthesis and Findings	Overall Risk of Bias
Jeffery, 2019 [2]	Low	Low	Low	Low	Low
Fuccio, 2019 [14]	Low	Low	Low	Low	Low
Shinkins, 2017 [13]	Low	Low	Low	Low	Low
Pita-Fernández, 2015 [12]	Low	Low	Low	Low	Low

**Table A4:** Risk of bias, RCTs.

Study	Domain 1: Randomization Process	Domain 2: Deviation from Intervention	Domain 3: Missing Outcome Data	Domain 4: Measurement of Outcome	Domain 5: Reported Result	Overall Risk of Bias
Wille-Jorgensen, 2018 [3]	Low/Some concerns	Low/Some concerns	Low	Low	Low	Low
Mant, 2017 FACS [4]	Low	Some concerns	Low	Low	Low	Low

Abbreviations: RCTs = randomized controlled trials.
